# Characteristics and Comparative Analysis of Mitochondrial Genomes of the Aphid Genus *Hyalopterus* Koch (Hemiptera: Aphididae: Aphidinae)

**DOI:** 10.3390/insects15060389

**Published:** 2024-05-27

**Authors:** Xiaolu Zhang, Cailing Li, Liyun Jiang, Gexia Qiao, Jing Chen

**Affiliations:** 1 Key Laboratory of Zoological Systematics and Evolution, Institute of Zoology, Chinese Academy of Sciences, Beijing 100101, China; zhangxiaolu@ioz.ac.cn (X.Z.); licailing@ioz.ac.cn (C.L.); jiangliyun@ioz.ac.cn (L.J.); 2 College of Life Sciences, University of Chinese Academy of Sciences, Beijing 100049, China

**Keywords:** mitogenome, insect, repeat region, phylogeny

## Abstract

**Simple Summary:**

*Hyalopterus* aphids pose a significant threat to stone fruit trees in the genus *Prunus* and are distributed worldwide. In this study, we generated and compared the mitochondrial genomes of three constituent species of *Hyalopterus*. The repeat region located between *trnE* and *trnF* exists but differs among the three species, and the repeat unit shows variation among different geographic populations within the widely distributed *Hyalopterus arundiniformis*. The phylogenetic trees based on complete mitochondrial genomes supported the monophyly of *Hyalopterus*, with *H. arundiniformis* placed at the basal position. Our study enriches the mitochondrial genomic library of Aphidinae and suggests the potential utility of repeat regions in intraspecific aphid diversification studies.

**Abstract:**

Using Illumina sequencing technology, we generated complete mitochondrial genomes (mitogenomes) of three constituent species of the aphid genus *Hyalopterus* Koch, *Hyalopterus amygdali* (Blanchard), *Hyalopterus arundiniformis* Ghulamullah, and *Hyalopterus pruni* (Geoffroy). The sizes of the *Hyalopterus* mitogenomes range from 15,306 to 15,410 bp, primarily due to variations in the length of non-coding regions. The *Hyalopterus* mitogenomes consist of 37 coding genes arranged in the order of the ancestral insect mitogenome, a control region, and a repeat region between *trnE* and *trnF.* According to the COI-based analysis, one previously reported mitogenome of *H. pruni* should be assigned to *H. arundiniformis*. The gene order, nucleotide composition, and codon usage in the *Hyalopterus* mitogenomes are highly conserved and similar to those of other species of Aphidinae. The tandem repeat units differ in nucleotide composition, length, and copy number across three *Hyalopterus* species. Within the widespread Eurasian species *H. arundiniformis*, variation in repeat units among different geographic populations is observed, indicating that the repeat region may provide valuable insights for studying the intraspecific diversification of aphids. Phylogenetic analyses based on 28 complete mitogenomes of Aphidinae supported the monophyly of Aphidinae, Aphidini, Macrosiphini, and two subtribes of Aphidini. *Hyalopterus* was monophyletic. *H. amygdali* and *H. pruni* formed a sister group, while *H. arundiniformis* was placed basally. Characterization of the mitogenomes of *Hyalopterus* provides valuable resources for further comparative studies and for advancing our understanding of the aphid mitogenome architecture.

## 1. Introduction

Aphids of the genus *Hyalopterus* Koch (Aphidinae: Aphidini) are widespread pests that cause severe damage to stone fruit crops of *Prunus* (Rosaceae) [1]. These species are characterized by elongated bodies, small siphunculi, and white powdery wax covering [1]. Recent studies, employing morphological and molecular techniques, have confirmed the existence of three species that alternate between different *Prunus* species and *Phragmites* within *Hyalopterus* [2,3,4,5,6,7]. *Hyalopterus amygdali* (Blanchard) feeds on the undersides of *Prunus dulcis* leaves and, occasionally, on *Prunus armeniaca* leaves. It migrates to reeds and possibly grasses as its secondary host plants. This species is mainly distributed in the Mediterranean area, central Asia, and possibly North America [1]. *Hyalopterus arundiniformis* Ghulamullah primarily feeds on *Prunus persica* and sometimes on *P. armeniaca*, *Prunus salicina,* and *Prunus cerasifera.* It is distributed throughout Eurasia [1]. *Hyalopterus pruni* (Geoffroy) inhabits the undersides of the leaves of *Prunus domestica* and sometimes is found on other *Prunus* species, except for *P. dulcis. H. pruni* is widely distributed in Europe and Asia and has been introduced to North America [1,8].

Due to its small size, maternal inheritance, fast evolutionary rate, and limited recombination, the insect mitogenome has been extensively employed in phylogenetic analysis across various taxonomic levels [9,10,11,12,13,14]. Compared to a single gene marker, the mitogenome provides more comprehensive information about the composition and arrangement of genes, which allows for a deeper understanding of the characteristics of species at the genetic level [10,15]. To date, complete mitogenomes of 27 aphid species of the subfamily Aphidinae have been sequenced and released on NCBI. The mitogenome of Aphidinae is a closed naked circular DNA, ranging from 15,111 to 17,954 bp in length, with a high A + T content ranging from 83.5% to 84.9%. The genus *Hyalopterus* belongs to the subtribe Rhopalosiphina of Aphidini. To date, complete mitogenomes are available for only five species of Rhopalosiphina [16,17,18].

In this study, we obtained the complete mitogenomes of *H. amygdali*, *H. arundiniformis*, and *H. pruni* via high-throughput sequencing to provide valuable mitogenomic information for Rhopalosiphina aphids. We compared the size, organization, nucleotide composition, codon usage, and non-coding regions of the *Hyalopterus* mitogenomes and constructed phylogenetic relationships of Aphidinae using maximum-likelihood and Bayesian methods based on the complete mitogenome sequences.

## 2. Materials and Methods

### 2.1. Sample Collection and DNA Extraction

Aphid specimens of the genus *Hyalopterus* were deposited in the National Animal Collection Resource Center, Institute of Zoology, Chinese Academy of Sciences, Beijing, China, and stored in 95% ethanol at −30 °C. Samples of *H. amygdali* were collected on *Phragmites australis* from Osh, Kyrgyzstan (voucher no. 40542); *H. arundiniformis* on *P. persica* from Fangchenggang, Guangxi, China (voucher no. AA197); and *H. pruni* on *P. australis* from Jambyl, Kazakhstan (voucher no. 47219). The slide-mounted voucher specimens were identified based on morphology using the taxonomic keys in Lozier et al. [5] and Rakauskas et al. [6]. The DNA was extracted from whole-body tissues using TRIzol^®^ Reagent (Invitrogen, Carlsbad, CA, USA).

### 2.2. Mitogenome Sequencing, Assembly, and Annotation

Mitogenome sequencing was carried out on an Illumina HiSeq 4000 platform (BIOZERON Co., Ltd., Shanghai, China), and quality control was performed with Trimmomatic-0.39 [19]. The scaffolds generated by *de novo* assembly with SPAdes v3.10.1 [20] were gap-filled and optimized using GapCloser v1.12 [21]. The preliminary annotation results were obtained by the MITOS2 [22] with the invertebrate mitochondrial genetic code and RefSeq 89 Metazoa as a reference. The annotation and secondary structures of transfer RNA genes (tRNAs) were predicted by MITOS. The ribosomal RNA genes (rRNAs) and protein-coding genes (PCGs) were annotated by aligning with the mitogenomes of related aphid species, and the PCGs were further validated using ORF Finder (https://www.ncbi.nlm.nih.gov/orffinder/, accessed on 5 January 2024). The circular maps of mitogenomes were visualized with GCView Server (https://proksee.ca/, accessed on 11 January 2024) [23].

### 2.3. Sequence Analyses

The nucleotide composition was determined by MEGA-X [24]. Nucleotide composition bias was measured by calculating AT skew = (A − T)/(A + T) and GC skew = (G − C)/(G + C) [25]. The relative synonymous codon usage (RSCU) of the PCGs was calculated and drawn using PhyloSuite v1.2.3pre3 [26]. The ratio of nonsynonymous substitution rate (Ka) to synonymous substitution rate (Ks) was calculated for each PCG using DnaSP 6 [27], with the mitogenome of *Pachypsylla venusta* (GenBank accession no. NC_006157) used as a reference [16,28]. The repeat region was detected through the Tandem Repeats Finder web server (https://tandem.bu.edu/trf/home, accessed on 19 January 2024) [29]. Secondary structures of the repeat unit and control region were folded using TBtools v1.064 [30] and drawn by VARNA v3-93 [31]. We calculated the sequence similarity of the repeat units in different mitogenomes using Sequence Manipulation Suite (Version 2) (https://www.detaibio.com/sms2/, accessed on 23 January 2024) [32].

### 2.4. Phylogenetic Analyses

The phylogenetic relationships of Aphidinae were inferred based on whole mitogenomes of 3 species from *Hyalopterus* and 24 other aphid species (Table S1) by maximum-likelihood (ML) approach and Bayesian inference (BI). Four species from Calaphidinae and Chaitophorinae were taken as outgroups based on previous phylogenetic studies [12,33,34,35]. The PCGs were aligned by MAFFT on the TranslatorX online server (http://translatorx.co.uk, accessed on 2 February 2024) [36]. We aligned the tRNA and rRNA genes with the MAFFT (version 7) online service (https://mafft.cbrc.jp/alignment/server/, accessed on 2 February 2024) [37,38] and removed the unreliable alignment regions using Gblocks 0.91b [39,40]. PartitionFinder2 [41] was used to evaluate the optimal partitioning scheme and substitution models on PhyloSuite v1.2.3pre3 [26] with linked branch lengths, BIC, and searching by the greedy algorithm. All 37 genes with a total of 14,352 bp were divided into six partitions. The ML analysis was inferred using RAxML v8.2.10 [42] with 1000 bootstrap replications and GTRGAMMAI model for each partition. The BI analysis was carried out by MrBayes 3.2.7 [43] with four chains, running for 2,000,000 generations. The sampling frequency was set to every 100 generations to obtain the average deviation of the split frequencies falling below 0.01. The first 25% trees were discarded as burn-in. The resulting phylogenetic trees were annotated by Interactive Tree Of Life (iTOL) v5 (https://itol.embl.de/, accessed on 16 February 2024) [44].

## 3. Results and Discussion

### 3.1. General Features of the Mitogenomes of Hyalopterus

**Figure 1:**
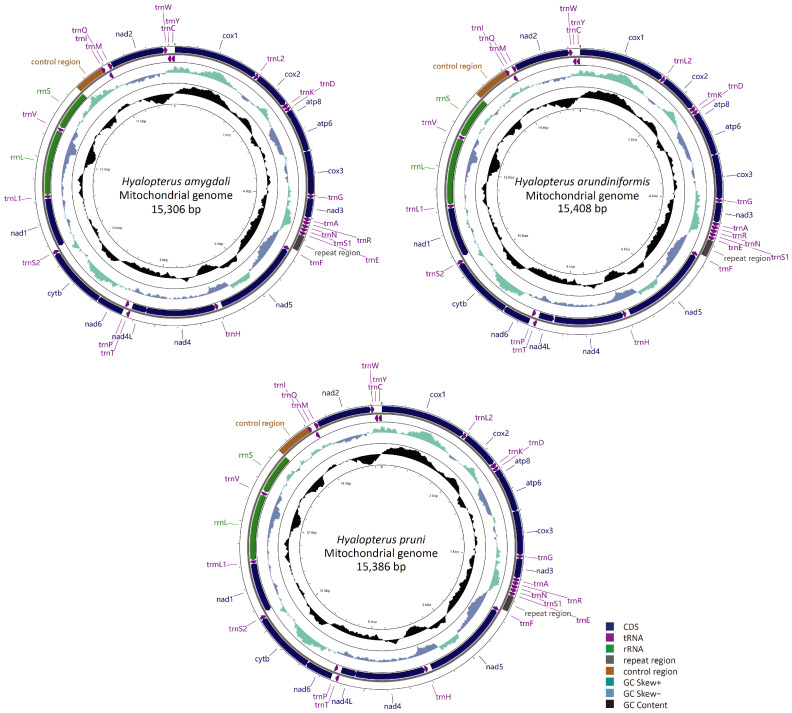
Circular maps of the mitogenomes of *Hyalopterus* species.

The complete mitogenome sequences of *H. amygdali, H. arundiniformis*, and *H. pruni* are closed circular molecules of 15,306 bp, 15,408 bp, and 15,386 bp in length, respectively (Figure 1). All sequences have been submitted to GenBank under accession numbers OK641613 (*H. amygdali*), OK274075 (*H. arundiniformis*), and OK641614 (*H. pruni*). The gene order of the *Hyalopterus* mitogenomes is conserved, following that of the putative ancestral insect mitogenome [45]. Each of the three mitogenomes contain 37 coding genes, including 13 PCGs, 22 tRNAs, and 2 rRNAs. Four PCGs, eight tRNAs, and two rRNAs are encoded by the minority strand (N strand), while the remaining twenty-three coding genes (nine PCGs and fourteen tRNAs) are located on the majority strand (J strand) (Figure 1, Table S2). The longest non-coding region in the mitogenome of *Hyalopterus*, known as the control region, is located between *rrnS* and *trnI*. Another long non-coding region is found between *trnE* and *trnF*, often referred to as the repeat region, which has been reported to be a unique feature of aphid mitogenomes [46,47,48].

There are ten overlaps and fourteen intergenic spacers in *H. amygdali*, ten overlaps and thirteen intergenic spacers in *H. arundiniformis*, and eleven overlaps and twelve intergenic spacers in *H. pruni* (Table S2). The longest gene overlap within the Aphidinae mitogenomes appears between *atp8* and *atp6*, ranging in size from 14 to 20 bp, except for *Aphis glycines* (NC_045236), which has a 1 bp intergenic spacer between *atp8* and *atp6* [47]. In the *Hyalopterus* mitogenomes, the longest overlap is 20 bp long and it is also located between *atp6* and *atp8*, similarly to most Aphidinae aphids. The largest intergenic spacer (63 bp) is located between *nad5* and *trnH*. Additionally, there is a 3 bp intergenic spacer between *trnR* and *trnN* in *H. amygdali*, but not in the other two species. In *H. arundiniformis* and *H. pruni*, a 2 bp overlap exists between *cob* and *trnS* (UCN), while *H. amygdali* has a 2 bp spacer between these two genes. There is a 7 bp overlap between *nad4* and *nad4L* in *H. amygdali* and *H. pruni* but an 8 bp intergenic spacer in *H. arundiniformis*.

The lengths of the mitogenomes reported in Aphididae range from 14 to 20 kb. The largest aphid mitogenome is 19,200 bp long in *Therioaphis tenera*, containing the largest control region of 1452 bp and a long repeat region [49,50]. The shortest mitogenome is 14,990 bp long in *Schizoneuraphis gallarum* and it has a compact gene organization [51]. The *Hyalopterus* mitogenomes all fall within the normal size range. The variable size of aphid mitogenomes is not closely related to the coding genes but is mainly due to differences among the non-coding regions, especially the control regions and repeat regions [28,48,50,52].

Liang et al. [17] reported the complete mitogenome of *H. pruni* under GenBank accession no. NC_050904. This aphid sample was collected from Ningxia, China. However, according to Liu et al. [7], *H. pruni* in China is only found in the northern Tianshan Mountains, and most common Chinese records of *H. pruni* are actually referring to *H. arundiniformis*. To confirm this, we constructed a neighbor-joining (NJ) tree with MEGA-X [24] based on the COI sequences obtained from Liu et al. [7] and the extracted COI sequences from NC_050904 and the mitogenome of *H. arundiniformis* sequenced in this study (voucher no. AA197). The NC_050904 and AA197 samples were both placed within the *H. arundiniformis* clade (Figure S4). Therefore, the mitogenome of *H. pruni* (NC_050904) reported by Liang et al. [17] should be assigned to *H. arundiniformis*.

### 3.2. Nucleotide Composition

The nucleotide composition of insect mitogenomes is generally unbalanced [16,53]. In the reported Aphidinae mitogenomes, the nucleotide compositions all display a strong bias toward A + T, with the A + T content ranging from 83.50% (*Aphis aurantii*, GenBank accession no. NC_052865) to 84.90% (*Uroleucon sonchi*, GenBank accession no. MT533446). The whole mitogenomes of *H. amygdali*, *H. arundiniformis*, and *H. pruni* exhibit A + T contents of 83.90%, 83.80% and 83.88%, respectively (Table 1). The A + T content of the repeat regions is 89.90%, 89.30%, and 88.38% in *H. amygdali*, *H. arundiniformis*, and *H. pruni*, respectively. The third codon position of the PCGs exhibits the highest A + T content (93.20–93.70%), while that of the other codon positions varies from 75.66% to 80.14% (Table 1). The whole mitogenome of *Hyalopterus* displays a slight bias toward A, with an AT-skew value of 0.07–0.08, and a moderate bias toward C, with GC-skew values ranging from −0.28 to −0.25 (Table 1). The PCGs encoded on the J strand are C-skewed, with GC-skew values ranging from −0.24 to −0.22, while the PCGs encoded on the N strand are G-skewed, with a GC-skew value of 0.30–0.35. These results indicate the presence of nucleotide composition heterogeneity between two strands in the aphid mitogenomes. The strand asymmetry may be attributed to the spontaneous deamination of the A and C nucleotides in the N strand during replication [53].

### 3.3. Protein-Coding Genes

The total lengths of thirteen PCGs in the mitogenomes of *H. amygdali*, *H. arundiniformis*, and *H. pruni* are 10,920 bp, 10,905 bp, and 10,920 bp, respectively. Nine of them (*cox1*, *cox2*, *atp8*, *atp6*, *cox3*, *nad3*, *nad6*, *cob*, and *nad2*) are located on the J strand, while the remaining four PCGs (*nad5*, *nad4*, *nad4L*, and *nad1*) are encoded by the N strand (Table S2). In aphid mitogenomes, most start codons are ATN and stop codons are TAA or TAG [28,48,50]. The start codons of the *Hyalopterus* mitogenomes are the same as those of the other aphids, which are ATA, ATT, and ATG, except for *nad3* in *H. arundiniformis*, which starts with ATC. In terms of stop codons, *cob* ends with TAG, while *cox1* and *nad4* end with a single T, which is common in insects and can be completed by post-transcriptional polyadenylation [54]. The remaining PCGs in the *Hyalopterus* mitogenomes all end with TAA (Table S2).

**Figure 2:**
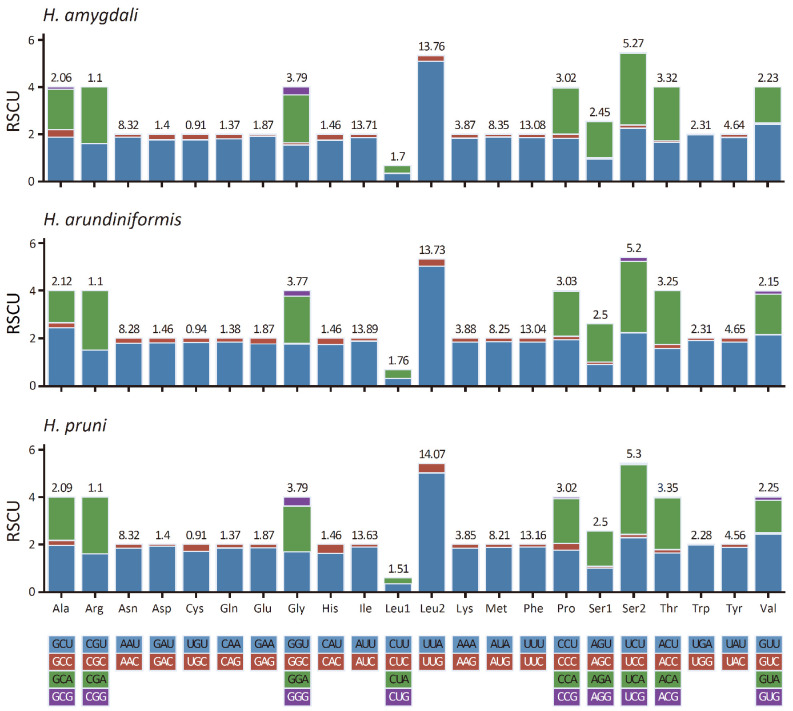
Relative synonymous codon usage (RSCU) in the mitogenomes of *Hyalopterus* species, with amino acid frequencies marked above the bars.

The codon usage of PCGs in the mitogenomes of *Hyalopterus* is shown in Figure 2 by RSCU. As in most aphids, the five most abundant codon families in the *Hyalopterus* mitogenomes are Phe, Ile, Leu (UUR), Met, and Asn, while Cys is the least abundant (Figure 2) [28]. Among the 51 amino acid-coding codons in the mitogenomes of *Hyalopterus*, the most frequently used codon is UUA(L), followed by AUU(I), UUU(F), AUA(M), and AAU(N). These frequently used codons are composed mainly of A or U nucleotides, which is consistent with the strong A + T-content bias of the whole mitogenomes in *Hyalopterus*.

**Figure 3:**
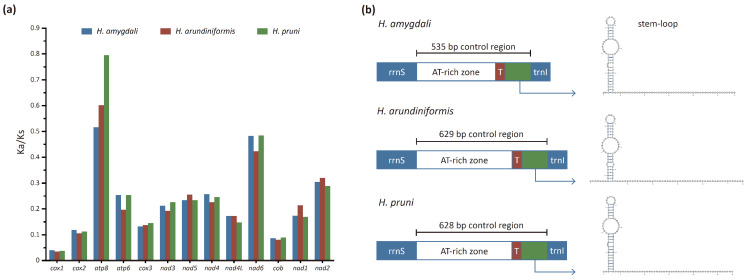
(**a**) Ka/Ks of thirteen protein-coding genes within the mitogenomes of *Hyalopterus* species. (**b**) Organization of the control regions and secondary structures of the stem-loop regions.

The Ka/Ks values of thirteen PCGs in the *Hyalopterus* mitogenomes are displayed in Figure 3a. All Ka/Ks values were below one, indicating that the primary evolutionary force shaping the *Hyalopterus* mitogenome sequences was purifying selection. The *atp8* gene showed the highest evolutionary rate, particularly in *H. pruni* with a value of 0.80, while in *H. amygdali* and *H. arundiniformis*, the values were 0.52 and 0.60, respectively. The second highest Ka/Ks values were observed for *nad6* (*H. amygdali*: 0.48; *H. arundiniformis*: 0.42; *H. pruni*: 0.48). The *cox1* gene exhibited the strongest purifying pressure with Ka/Ks values of 0.04, 0.03, and 0.04 in *H. amygdali*, *H. arundiniformis*, and *H. pruni*, respectively (Figure 3a).

### 3.4. Transfer and Ribosomal RNA Genes

Each mitogenome of the three *Hyalopterus* species contains 22 tRNAs, ranging from 63 to 73 bp in size (Table S2). All tRNAs can form the typical clover-leaf secondary structures except for *trnS* (AGN), which lost the dihydrouridine (DHU) arm (Figures S1–S3). This feature is commonly observed in aphid mitogenomes.

The mitogenomes of *Hyalopterus* species encode two rRNAs on the N strand, both of which are in typical locations and have typical sizes for aphid mitogenomes (Table S2). The *rrnL* gene is located between *trnL* (CUN) and *trnV* without any overlapping or intergenic spacers. In *H. amygdali* and *H. pruni*, *rrnL* is 1257 bp long, while in *H. arundiniformis*, it is 1260 bp long. The lengths of *rrnS* are 770 bp, 768 bp, and 767 bp in *H. arundiniformis*, *H. amygdali*, and *H. pruni*, respectively. There is an intergenic spacer of 12 or 13 bp between *trnV* and the control region (12 bp in *H. amygdali* and *H. arundiniformis*, and 13 bp in *H. pruni*).

### 3.5. Control Region

The control region is a long non-coding region characterized by an extremely high A + T content in aphid mitogenomes. In Hemiptera insects, the A + T content of the control region ranges from 63.87% to 93.02% [50]. In *H. amygdali*, *H. arundiniformis*, and *H. pruni*, the A + T contents of the control region are 84.10%, 85.53%, and 86.31%, respectively (Table 1). The control region is involved in the initiation of DNA replication and contains promoters for the transcription of both mitochondrial DNA strands [46,55,56]. It is located between *trnI* and *rrnS* in the *Hyalopterus* mitogenome, and consists of an AT-rich zone, a poly-thymidine stretch, and a stem-loop region, which can be folded into a stable secondary structure (Figure 3b).

The control region exhibits poor conservation among *Hyalopterus* species due to high rates of nucleotide substitution and indels [52,55]. The lengths of the control regions are 535 bp, 629 bp, and 628 bp in *H. amygdali*, *H. arundiniformis*, and *H. pruni*, respectively (Table S2). The control region of *H. amygdali* is notably shorter than those of the other two species, possibly due to its shorter AT-rich zone of 413 bp. In comparison, the AT-rich zones of *H. arundiniformis* and *H. pruni* are 508 bp and 507 bp long, respectively.

### 3.6. Repeat Region

**Figure 4:**
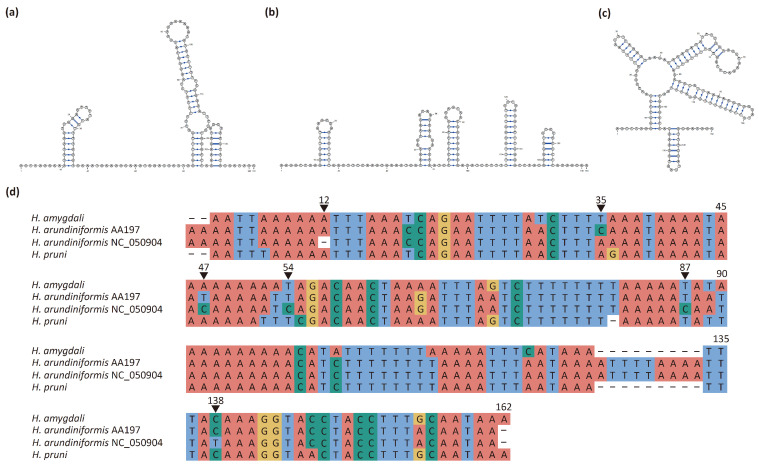
(**a**–**c**) Putative stem-loop structures of the repeat units of *H. amygdali* (**a**), *H. arundiniformis* (**b**), and *H. pruni* (**c**). (**d**) Pairwise alignment of the repeat units of three *Hyalopterus* mitogenomes sequenced in the present study and the previously reported one (NC_050904).

The repeat region in aphid mitogenomes is a tandem repeat sequence that is commonly found between *trnE* and *trnF* and sometimes within the control region [28,46,48,50]. The repeat region located between *trnE* and *trnF* is surmised to serve as another origin for replication [46,47]. It is hypothesized to have originated in the common ancestor of Aphididae and subsequently experienced numerous losses during species diversification [47,48,57]. In the *Hyalopterus* mitogenomes, the repeat region is only present between *trnE* and *trnF* and it exhibits the highest A + T content compared to other regions (*H. amygdali*: 89.90%; *H. arundiniformis*: 89.30%; *H. pruni*: 88.38%) (Table 1). *H. amygdali* has a repeat region of 286 bp in length, containing 1.834 151 bp-long repeat units; in *H. arundiniformis*, the repeat region is 299 bp in length and contains 1.832 161 bp-long repeat units; and the repeat region in *H. pruni* is 284 bp long, containing 1.860 150 bp-long repeat units (Figure 4d, Table 2). Each repeat unit can fold into a stem-loop structure (Figure 4a–c). The sequence similarity of the repeat units among the three species of *Hyalopterus* ranged from 82.10% to 91.39% (Table 2), which aligns with previous findings that tandem repeats exhibit high sequence similarity among closely related species [48].

The repeat unit of *H. arundiniformis* sequenced in the present study (voucher no. AA197) has a sequence similarity of 96.27% with the reported one (NC_050904) (Table 2). The repeat region is 299 bp long in AA197 and 298 bp long in NC_050904, containing 1.832 repeat units of 161 bp and 1.838 repeat units of 160 bp, respectively. In the repeat unit of AA197, the bases at positions 35, 47, 54, 87, and 138 are C, T, T, T, and C, respectively, while in the same position of NC_050904, the bases are A, C, C, C, and T, respectively (Figure 4d). Furthermore, compared with AA197, NC_050904 has a single nucleotide deletion at position 12 (Figure 4d). It has been reported that high nucleotide substitution rates, insertions or deletions, and variations in copy number are the factors that contribute to the intraspecific differences in non-coding regions of insect mitogenomes [56,58].

*H. arundiniformis* is a widespread Eurasian species [1]. To determine whether the variation in the repeat region of the two samples of *H. arundiniformis* was caused by geographical isolation, we constructed an ML tree using COI sequences of *H. arundiniformis* from AA197, NC_050904, and Liu et al. [59] (Figure S5). The AA197 sample collected from Guangxi, China was clustered within clade AL4, which was mainly composed of populations from southeastern and southwestern China [59]. The NC_050904 sample collected from Ningxia, China was nested within clade AL6, which spanned the Eurasia continent [59]. Therefore, the two samples of *H. arundiniformis* belong to distinct genetic lineages, and the intraspecific variation in the repeat region reported here may be applicable to aphid population genetic studies.

### 3.7. Phylogenetic Analyses

**Figure 5:**
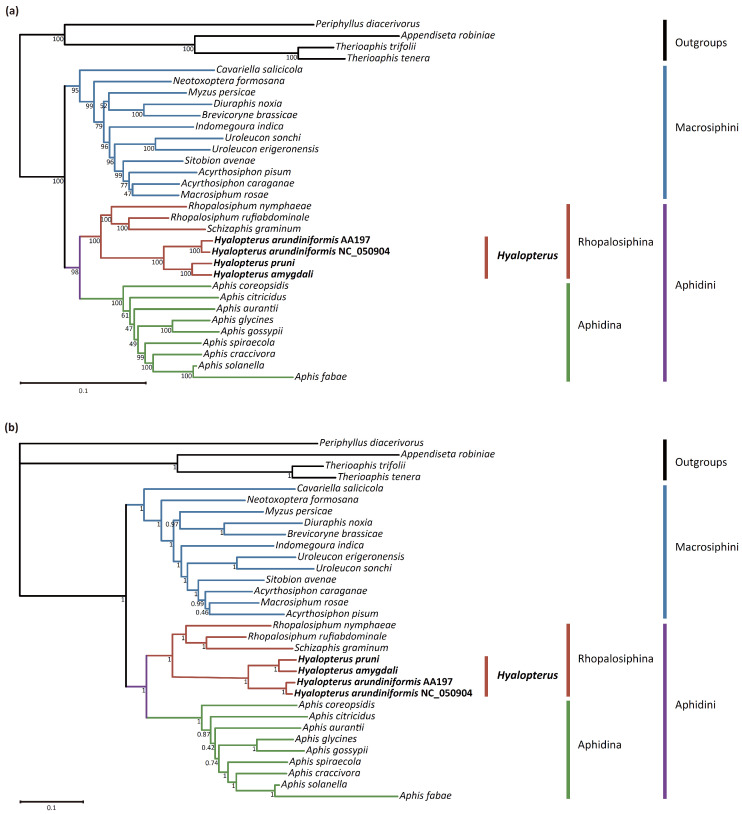
Aphidinae phylogenetic trees inferred from complete mitochondrial genomes. (**a**) Maximum-likelihood (ML) tree. The values under the branches indicate ML bootstrap probabilities. (**b**) Bayesian tree. The values under the branches indicate posterior probabilities.

To determine the phylogenetic relationships among aphid species of Aphidinae, we performed ML and BI phylogenetic analyses with complete mitogenome sequences. The ML and BI trees were almost congruent in topology (Figure 5). Both trees supported the monophyly of Aphidinae, two tribes (Aphidini and Macrosiphini), and two subtribes within Aphidini (Aphidina and Rhopalosiphina). The generic relationships within Macrosiphini were in line with those reported by Choi et al. [60]. *Cavariella* and *Neotoxoptera* were the earliest and second earliest diverging branches, respectively. *Myzus*, *Brevicoryne*, and *Diuraphis* were clustered together, and *Uroleucon*, *Sitobion*, *Acyrthosiphon*, and *Macrosiphum* formed a well-supported clade.

Within the Rhopalosiphina clade, the genus *Hyalopterus* was monophyletic with strong support (bootstrap, BS = 100%; posterior probability, PP = 1) and was placed as a sister to the clade of *Rhopalosiphum* and *Schizaphis*, which was consistent with previous research [61]. The reported mitogenome NC_050904 strongly grouped with *H. arundiniformi* (AA197) (BS = 100%, PP = 1), which was in line with the result of the COI NJ tree (Figure S4) and supported the conclusion that NC_050904 should be assigned to *H. arundiniformi*. *H. amygdali* was clustered with *H. pruni* (BS = 100%; PP = 1), while *H. arundiniformis* was placed basally. The interspecific phylogenetic relationship of *Hyalopterus* based on whole mitogenome sequences was consistent with previous studies using multiple genes [4,7].

## 4. Conclusions

Complete mitogenomes of the aphid genus *Hyalopterus*, including three species, *H. amygdali*, *H. arundiniformis*, and *H. pruni*, were obtained using next-generation sequencing technology. The *Hyalopterus* mitogenomes show high conservation in terms of gene order, nucleotide composition, and codon usage, as observed in other Aphidinae mitogenomes. The repeat region is variable in length and nucleotide sequence among the three species, and intraspecific variation in the repeat unit is found in *H. arundiniformis*. Phylogenetic analysis based on the Aphidinae mitogenomes demonstrated the usefulness of the mitogenomes in resolving the phylogenetic relationships of tribes and genera. These newly produced mitogenome sequences of *Hyalopterus* are valuable data resources for the study of Aphidinae aphids. The acquisition of more aphid mitogenomes is necessary to provide additional mitogenome characteristics and phylogenetic information to enhance our understanding of the phylogeny and evolution of aphids.

## Figures and Tables

**Table 1:** A + T content, AT-skew, and GC-skew of the *Hyalopterus* mitogenomes.

	*H. amygdali*			*H. arundiniformis*			*H. pruni*		
	A + T%	AT-Skew	GC-Skew	A + T%	AT-Skew	GC-Skew	A + T%	AT-Skew	GC-Skew
Whole genome	83.90	0.08	−0.25	83.80	0.08	−0.27	83.88	0.07	−0.27
PCGs	83.10	−0.15	−0.05	82.95	−0.15	−0.04	83.07	−0.16	−0.03
First codon	80.00	0	0.13	79.92	0.00	0.13	80.14	−0.01	0.15
Second codon	75.90	−0.40	−0.13	75.79	−0.39	−0.12	75.66	−0.40	−0.13
Third codon	93.70	−0.09	−0.29	93.20	−0.10	−0.22	93.41	−0.10	−0.17
PCGs-J	82.00	−0.06	−0.23	81.74	−0.06	−0.23	81.99	−0.07	−0.22
PCGs-J-first codon	79.20	0.12	0	78.66	0.12	−0.01	79.11	0.11	0.01
PCGs-J-second codon	73.80	−0.36	−0.27	73.71	−0.36	−0.26	73.53	−0.36	−0.27
PCGs-J-third codon	93.40	0.015	−0.88	92.86	0.02	−0.80	93.35	0.00	−0.76
PCGs-N	85.00	−0.29	0.32	84.90	−0.30	0.35	84.79	−0.30	0.34
PCGs-N-first codon	81.40	−0.18	0.38	81.50	−0.18	0.40	81.79	−0.19	0.40
PCGs-N-second codon	79.60	−0.46	0.15	78.70	−0.45	0.15	79.07	−0.46	0.15
PCGs-N-third codon	94.40	−0.25	0.69	93.50	−0.28	0.84	93.50	−0.27	0.80
tRNA genes	85.60	0.04	0.18	85.77	0.04	0.19	85.50	0.05	0.13
rRNA genes	84.80	−0.07	0.34	84.93	−0.08	0.34	84.93	−0.07	0.34
Control region	84.10	−0.07	−0.16	85.53	−0.05	−0.32	86.31	−0.08	−0.30
Repeat region	89.90	0.19	−0.39	89.30	0.18	−0.50	88.38	0.14	−0.33

**Table 2:** Sequence information for the repeat regions of *Hyalopterus* mitogenomes.

Species	Length of Repeat Region (bp)	Length of Repeat Unit (bp)	Copy Number	Sequence Similarity of Repeat Unit (%)			
				*H. amygdali*	*H. arundiniformis* AA197	*H. arundiniformis* NC_050904	*H. pruni*
*H. amygdali*	286	151	1.834	–	84.57	82.10	91.39
*H. arundiniformis* AA197	299	161	1.832	84.57	–	96.27	84.57
*H. arundiniformis* NC_050904	298	160	1.838	82.10	96.27	–	82.72
*H. pruni*	284	150	1.860	91.39	84.57	82.72	–

## Data Availability

The data that support the findings of this study are openly available in GenBank with accession numbers OK641613, OK274075, and OK641614.

## Supplementary Materials

The following supporting information can be downloaded at: https://www.mdpi.com/article/10.3390/insects15060389/s1, Figure S1: Secondary structures of 22 transfer RNAs in *H. amygdali*, Figure S2: Secondary structures of 22 transfer RNAs in *H. arundiniformis*, Figure S3: Secondary structures of 22 transfer RNAs in *H. pruni*, Figure S4: Neighbor-joining tree based on the COI sequences obtained from Liu et al. [7] and the extracted COI sequences from NC_050904 and the mitogenome of *H. arundiniformis* sequenced in this study (voucher no. AA197), Figure S5: Maximum-likelihood tree constructed based on COI sequences of *H. arundiniformis* from Liu et al. [59], AA197, and NC_050904, using IQ-TREE v2.2.0 with the “TEST” model and 5000 bootstrap replications. Clades were numbered following Liu et al. [59], Table S1: Complete mitogenomes of aphids used for phylogenetic analysis, Table S2: Mitogenome organizations of three *Hyalopterus* species.
