# Supplementary material for: Characteristics and Comparative Analysis of Mitochondrial Genomes of the Aphid Genus Hyalopterus Koch (Hemiptera: Aphididae: Aphidinae)
Source: Insects. 2024 May 27;15(6):389. doi: 10.3390/insects15060389 (PMC11204073; doi:10.3390/insects15060389)
Supplement: Supplementary file 1 [file insects-15-00389-s001.zip › insects-3001293-supplementary.pdf]

Supplementary materials

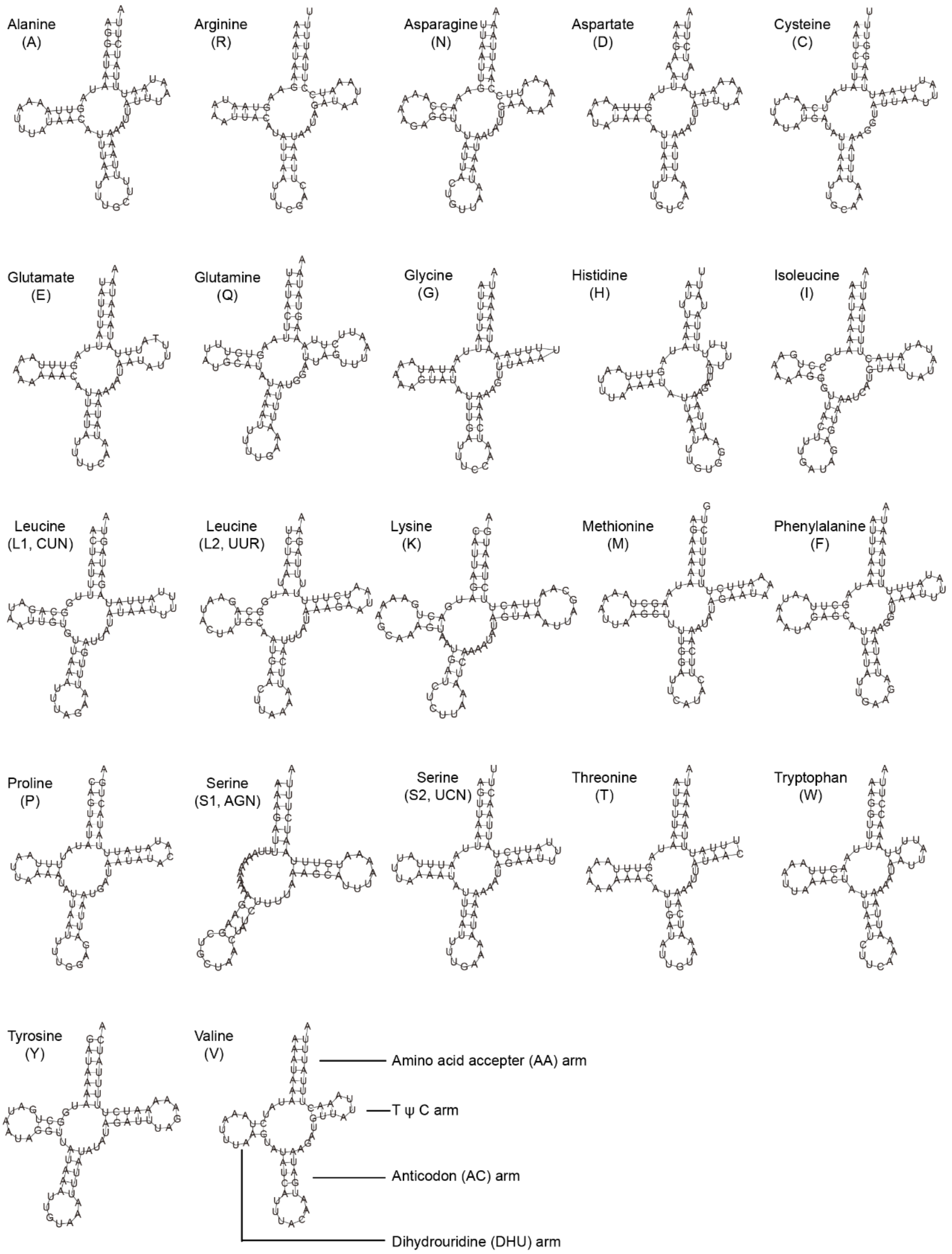

Figure S1. Secondary structures of 22 transfer RNAs in *H. amygdali*.

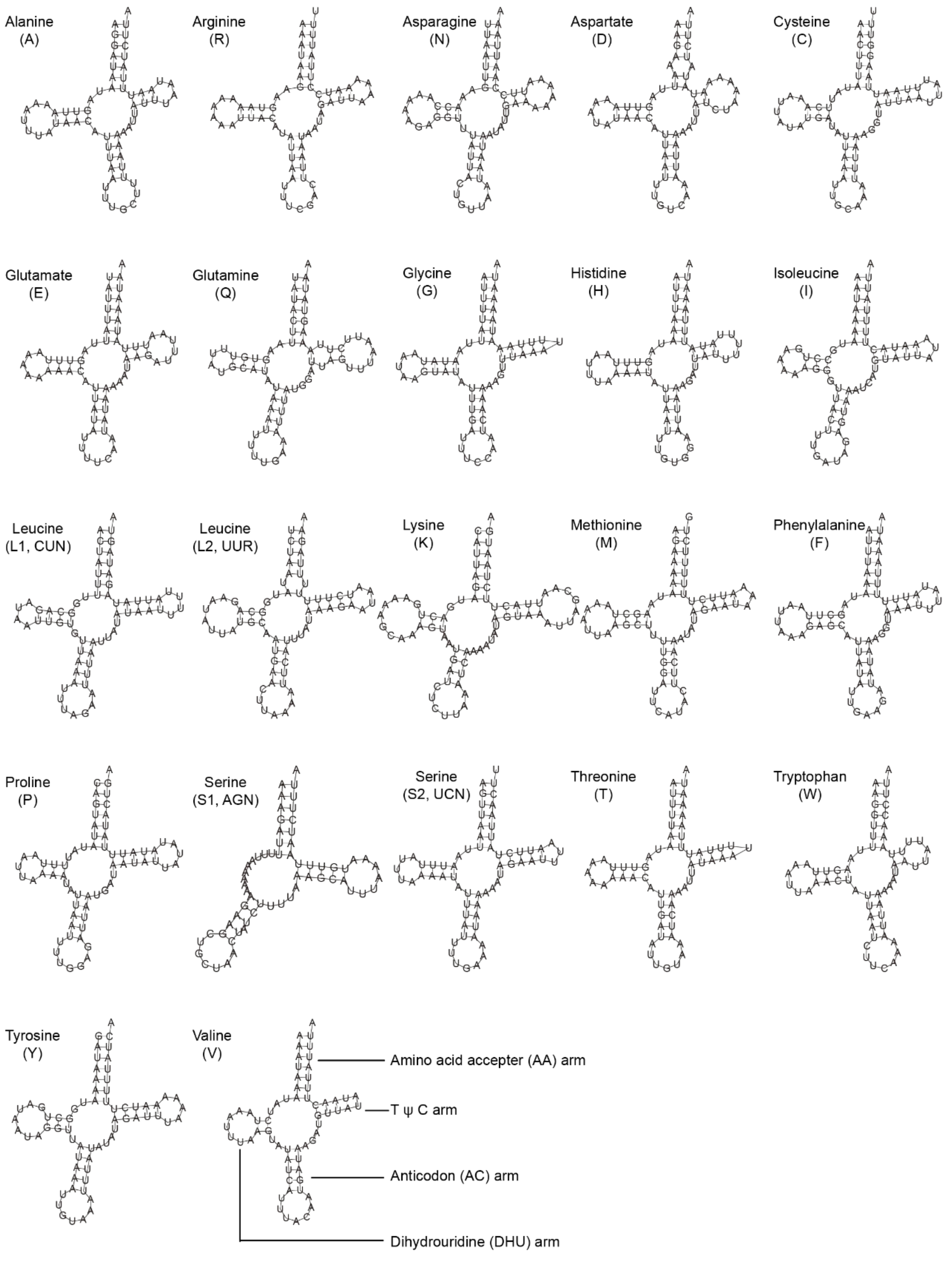

Figure S2. Secondary structures of 22 transfer RNAs in *H. arundiniformis*.

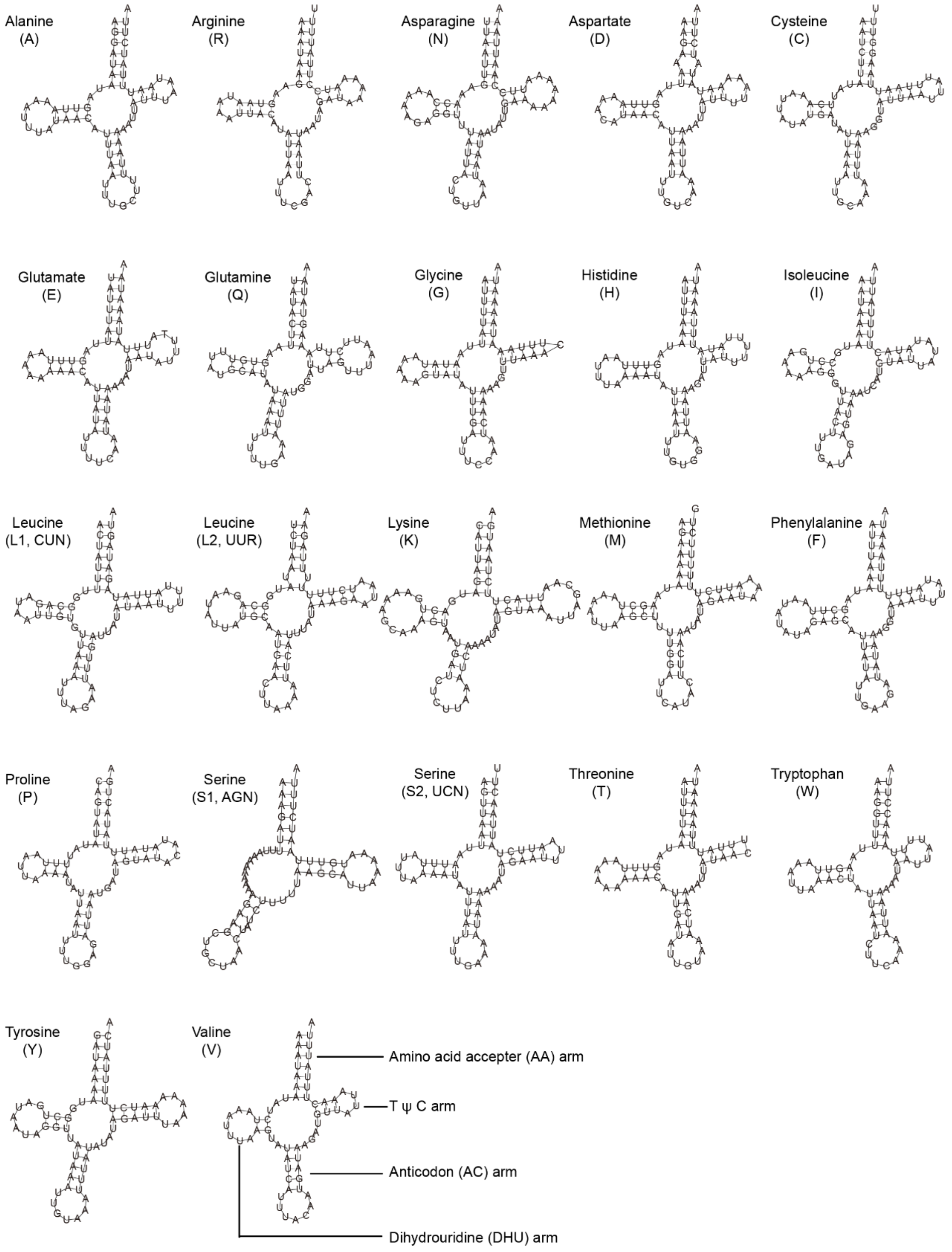

Figure S3. Secondary structures of 22 transfer RNAs in *H. pruni*.

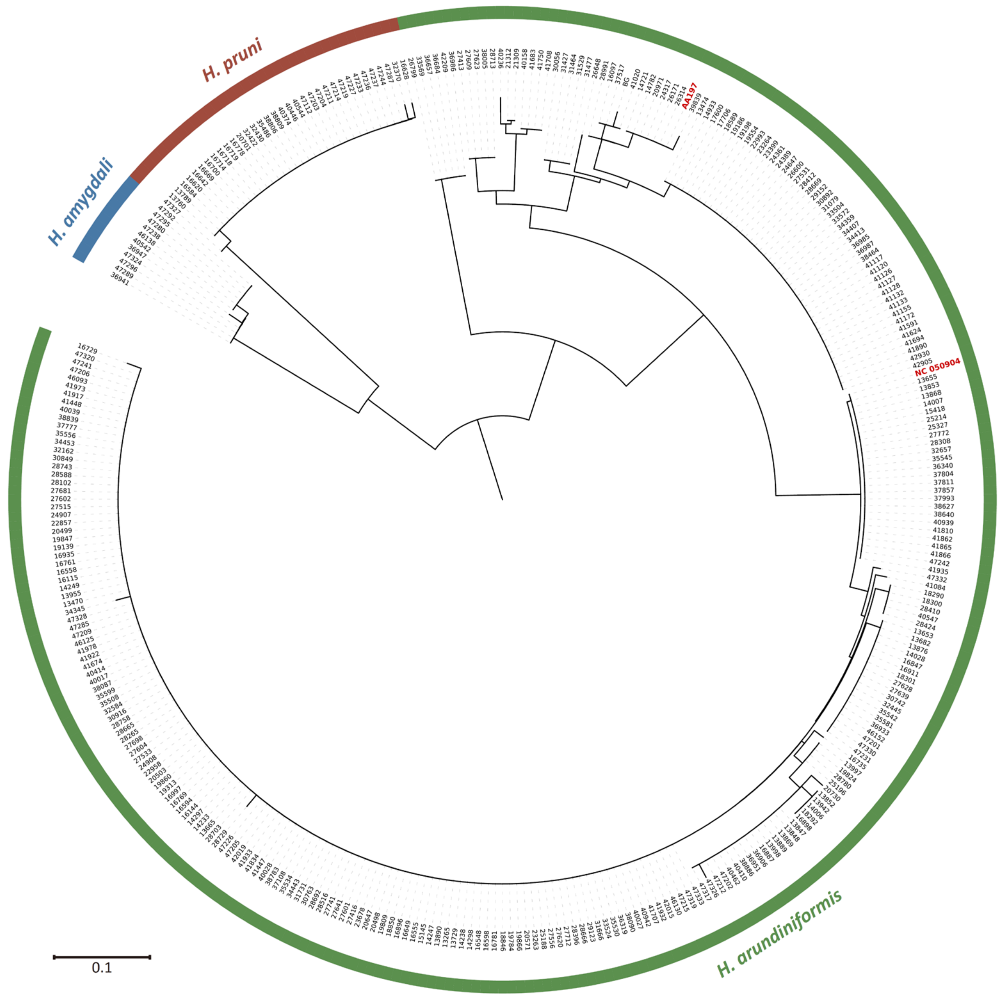

Figure S4. Neighbor-joining tree based on the COI sequences obtained from Liu et al. [7] and the extracted COI sequences from NC_050904 and the mitogenome of *H. arundiniformis* sequenced in this study (voucher no. AA197).

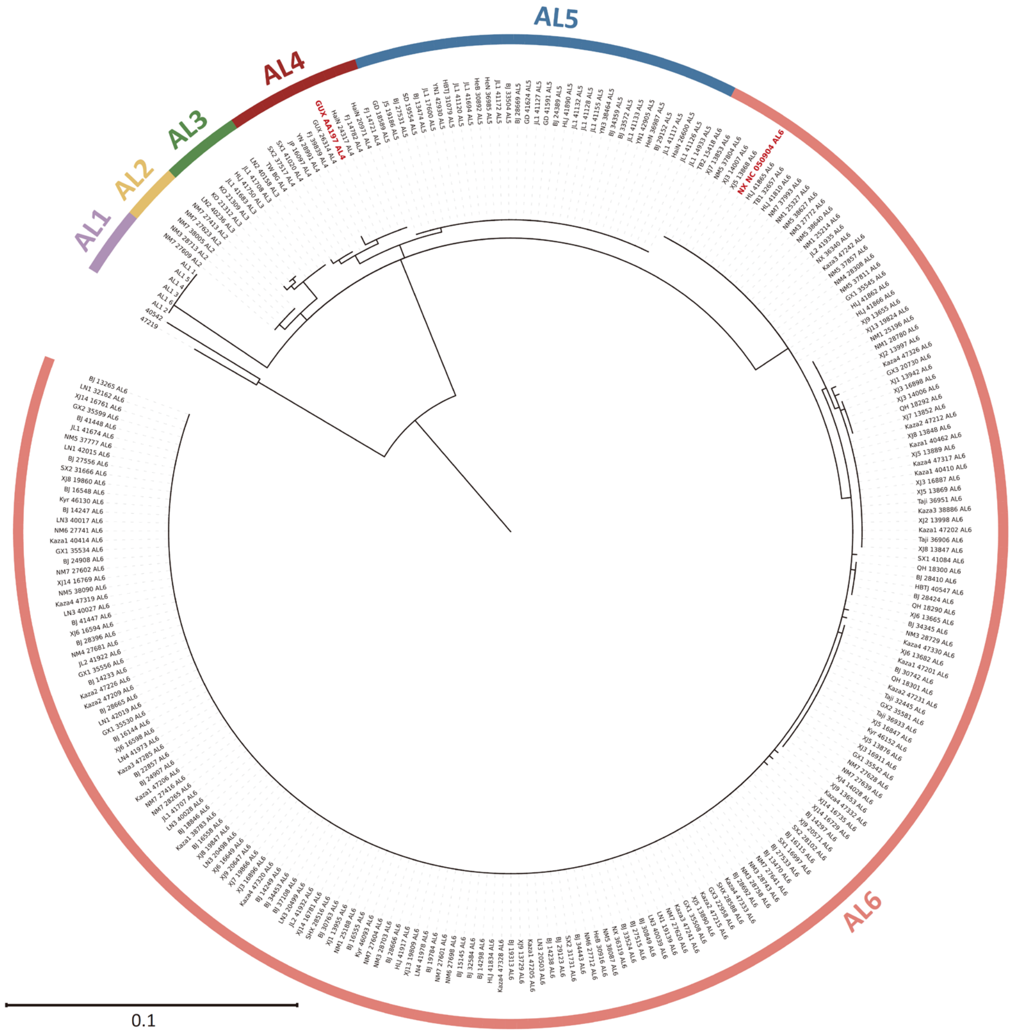

Figure S5. Maximum-likelihood tree constructed based on COI sequences of *H. arundiniformis* from Liu et al. [59], AA197, and NC_050904, using IQ-TREE v2.2.0 with the "TEST" model and 5000 bootstrap replications. Clades were numbered following Liu et al. [59].

Table S1. Complete mitogenomes of aphids used for phylogenetic analysis.

Subfamily	Tribe	Subtribe	Species	Length (bp)	Accession Number
Aphidinae	Aphidini	Aphidina	*Aphis* (*Toxoptera*) *aurantii* Boyer de Fonscolombe, 1841	15296	NC_052865
			Aphis craccivora Koch, 1854	15308	NC_031387
			Aphis (*Toxoptera*) *citricidus* (Kirkaldy, 1907)	16763	NC_043903
			Aphis (*Aphis*) *coreopsidis* (Thomas, 1878)	15623	NC_068763
			Aphis fabae mordvilkoi Börner & Janich, 1922	15346	NC_039988
			Aphis glycines Matsumura, 1917	17954	NC_045236
			Aphis gossypii Glover, 1877	15869	NC_024581
			Aphis (Aphis) solanella Theobald, 1914	15331	NC_068764
			Aphis (Aphis) spiraecola Patch, 1914	15465	NC_053819
		Rhopalosiphina	*Hyalopterus amygdali* (Blanchard, 1840)	15306	OK641613
			Hyalopterus arundiniformis Ghulamullah, 1942	15408	OK274075
			Hyalopterus arundiniformis Ghulamullah, 1942	15410	NC_050904
			Hyalopterus pruni (Geoffroy, 1762)	15386	OK641614
			Rhopalosiphum nymphaeae (Linnaeus, 1761)	15594	MN943499
			Rhopalosiphum rufiabdominale (Sasaki, 1899)	15289	NC_062327
			Schizaphis graminum (Rondani, 1852)	15721	NC_006158
	Macrosiphini		*Acyrthosiphon pisum* (Harris, 1776)	16971	NC_011594
			Acyrthosiphon (Acyrthosiphon) caraganae (Cholodkovsky, 1908)	15933	NC_064371
			Brevicoryne brassicae (Linnaeus, 1758)	15749	MW267824
			Cavariella salicicola (Matsumura, 1917)	16317	NC_022682
			Diuraphis noxia (Mordvilko, 1913)	15784	NC_022727
			Indomegoura indica (van der Goot, 1916)	15220	NC_045897
			Macrosiphum (*Macrosiphum*) *rosae* (Linnaeus, 1758)	15200	NC_064372
			Myzus (*Nectarosiphon*) *persicae* (Sulzer, 1776)	17382	NC_029727
			Neotoxoptera formosana (Takahashi, 1921)	15642	NC_057970

**Subfamily**	**Tribe**	**Subtribe**	**Species**	**Length (bp)**	**Accession Number**
Calaphidinae	Panaphidini	Panaphidina	*Sitobion avenae* (Fabricius, 1775)	15180	NC_024683
			Uroleucon erigeronensis (Thomas, 1878)	15691	MZ695840
			Uroleucon sonchi (Linnaeus, 1767)	17271	MT533446
			Appendiseta robiniae (Gillette, 1907)	15049	MH643884
			Therioaphis tenera (Aizenberg, 1956)	19200	MH643885
			Therioaphis (*Pterocallidium*) *trifolii* (Monell, 1882)	16068	MK766411
Chaitophorinae	Chaitophorini		*Periphyllus diacerivorus* Zhang, 1982	16418	MZ665537

Table S2. Mitogenome organizations of three *Hyalopterus* species.

Gene	Species	Strand	Start	End	Size (bp)	Anticodon	Start Codon	Stop Codon	Intergenic Nucleotides (bp)
cox1	*H. amygdali*	J	1	1531	1531		ATA	T	0
	H. arundiniformis		1	1531	1531		ATA	T	0
	H. pruni		1	1531	1531		ATA	T	0
trnL2	*H. amygdali*	J	1532	1598	67	TAA			3
	H. arundiniformis		1532	1598	67	TAA			3
	H. pruni		1532	1598	67	TAA			3
cox2	*H. amygdali*	J	1602	2273	672		ATA	TAA	2
	H. arundiniformis		1602	2273	672		ATA	TAA	2
	H. pruni		1602	2273	672		ATA	TAA	2
trnK	*H. amygdali*	J	2276	2348	73	CTT			0
	H. arundiniformis		2276	2348	73	CTT			0
	H. pruni		2276	2348	73	CTT			0
trnD	*H. amygdali*	J	2349	2411	63	GTC			0
	H. arundiniformis		2349	2411	63	GTC			0
	H. pruni		2349	2411	63	GTC			0
atp8	*H. amygdali*	J	2412	2570	159		ATT	TAA	–20
	H. arundiniformis		2412	2570	159		ATT	TAA	–20
	H. pruni		2412	2570	159		ATT	TAA	–20
atp6	*H. amygdali*	J	2551	3204	654		ATT	TAA	–1
	H. arundiniformis		2551	3204	654		ATT	TAA	–1
	H. pruni		2551	3204	654		ATT	TAA	–1
cox3	*H. amygdali*	J	3204	3989	786		ATG	TAA	–1
	H. arundiniformis		3204	3989	786		ATG	TAA	–1
	H. pruni		3204	3989	786		ATG	TAA	–1
trnG	*H. amygdali*	J	3989	4051	63	TCC			0
	H. arundiniformis		3989	4051	63	TCC			0

Gene	Species	Strand	Start	End	Size (bp)	Anticodon	Start Codon	Stop Codon	Intergenic Nucleotides (bp)
	H. pruni		3989	4051	63	TCC			0
nad3	*H. amygdali*	J	4052	4405	354		ATT	TAA	0
	H. arundiniformis		4052	4405	354		ATC	TAA	0
	H. pruni		4052	4405	354		ATT	TAA	0
trnA	*H. amygdali*	J	4406	4469	64	TGC			–1
	H. arundiniformis		4406	4469	64	TGC			–1
	H. pruni		4406	4469	64	TGC			–1
trnR	*H. amygdali*	J	4469	4531	63	TCG			3
	H. arundiniformis		4469	4534	66	TCG			0
	H. pruni		4469	4531	63	TCG			0
trnN	*H. amygdali*	J	4535	4600	66	GTT			–1
	H. arundiniformis		4535	4599	65	GTT			–1
	H. pruni		4532	4597	66	GTT			–1
trnS1	*H. amygdali*	J	4600	4663	64	GCT			2
	H. arundiniformis		4599	4661	63	GCT			2
	H. pruni		4597	4660	64	GCT			2
trnE	*H. amygdali*	J	4666	4729	64	TTC			0
	H. arundiniformis		4664	4727	64	TTC			0
	H. pruni		4663	4726	64	TTC			0
Repeat region	*H. amygdali*		4730	5015	286				0
	H. arundiniformis		4728	5026	299				0
	H. pruni		4727	5010	284				0
trnF	*H. amygdali*	N	5016	5082	67	GAA			0
	H. arundiniformis		5027	5092	66	GAA			0
	H. pruni		5011	5077	67	GAA			0
nad5	*H. amygdali*	N	5083	6741	1659		ATT	TAA	63
	H. arundiniformis		5093	6751	1659		ATT	TAA	63
	H. pruni		5078	6736	1659		ATT	TAA	63

Gene	Species	Strand	Start	End	Size (bp)	Anticodon	Start Codon	Stop Codon	Intergenic Nucleotides (bp)
trnH	*H. amygdali*	N	6805	6870	66	GTG			0
	H. arundiniformis		6815	6878	64	GTG			0
	H. pruni		6800	6863	64	GTG			0
nad4	*H. amygdali*	N	6871	8194	1324		ATG	T	–7
	H. arundiniformis		6879	8187	1309		ATA	T	8
	H. pruni		6864	8187	1324		ATG	T	–7
nad4L	*H. amygdali*	N	8188	8478	291		ATA	TAA	1
	H. arundiniformis		8196	8486	291		ATA	TAA	1
	H. pruni		8181	8471	291		ATA	TAA	1
trnT	*H. amygdali*	J	8480	8542	63	TGT			2
	H. arundiniformis		8488	8550	63	TGT			2
	H. pruni		8473	8535	63	TGT			2
trnP	*H. amygdali*	N	8545	8610	66	TGG			1
	H. arundiniformis		8553	8619	67	TGG			1
	H. pruni		8538	8603	66	TGG			1
nad6	*H. amygdali*	J	8612	9106	495		ATT	TAA	–1
	H. arundiniformis		8621	9115	495		ATT	TAA	–1
	H. pruni		8605	9099	495		ATT	TAA	–1
cob	*H. amygdali*	J	9106	10221	1116		ATG	TAG	2
	H. arundiniformis		9115	10230	1116		ATG	TAG	–2
	H. pruni		9099	10214	1116		ATG	TAG	–2
trnS2	*H. amygdali*	J	10224	10288	65	TGA			10
	H. arundiniformis		10229	10293	65	TGA			10
	H. pruni		10213	10277	65	TGA			10
nad1	*H. amygdali*	N	10299	11234	936		ATT	TAA	0
	H. arundiniformis		10304	11239	936		ATT	TAA	0
	H. pruni		10288	11223	936		ATT	TAA	0
trnL1	*H. amygdali*	N	11235	11299	65	TAG			0

Gene	Species	Strand	Start	End	Size (bp)	Anticodon	Start Codon	Stop Codon	Intergenic Nucleotides (bp)
	H. arundiniformis		11240	11304	65	TAG			0
	H. pruni		11224	11288	65	TAG			0
rrnL	*H. amygdali*	N	11300	12556	1257				0
	H. arundiniformis		11305	12564	1260				0
	H. pruni		11289	12545	1257				0
trnV	*H. amygdali*	N	12557	12618	62	TAC			12
	H. arundiniformis		12565	12626	62	TAC			12
	H. pruni		12546	12607	62	TAC			13
rrnS	*H. amygdali*	N	12631	13398	768				0
	H. arundiniformis		12639	13408	770				0
	H. pruni		12621	13387	767				0
Control region	*H. amygdali*		13399	13933	535				0
	H. arundiniformis		13409	14037	629				0
	H. pruni		13388	14015	628				0
trnI	*H. amygdali*	J	13934	13999	66	GAT			–3
	H. arundiniformis		14038	14101	64	GAT			–3
	H. pruni		14016	14079	64	GAT			–3
trnQ	*H. amygdali*	N	13997	14062	66	TTG			8
	H. arundiniformis		14099	14164	66	TTG			9
	H. pruni		14077	14142	66	TTG			8
trnM	*H. amygdali*	J	14071	14137	67	CAT			0
	H. arundiniformis		14174	14240	67	CAT			0
	H. pruni		14151	14217	67	CAT			0
nad2	*H. amygdali*	J	14138	15115	978		ATA	TAA	–2
	H. arundiniformis		14241	15218	978		ATA	TAA	–2
	H. pruni		14218	15195	978		ATA	TAA	–2
trnW	*H. amygdali*	J	15114	15175	62	TCA			–8
	H. arundiniformis		15217	15278	62	TCA			–8

Gene	Species	Strand	Start	End	Size (bp)	Anticodon	Start Codon	Stop Codon	Intergenic Nucleotides (bp)
	H. pruni		15194	15255	62	TCA			–8
trnC	*H. amygdali*	N	15168	15235	68	GCA			2
	H. arundiniformis		15271	15338	68	GCA			2
	H. pruni		15248	15315	68	GCA			2
trnY	*H. amygdali*	N	15238	15305	68	GTA			1
	H. arundiniformis		15341	15407	67	GTA			1
	H. pruni		15318	15385	68	GTA			1
